# Supplementary material for: A Machine-Generated View of the Role of Blood Glucose Levels in the Severity of COVID-19
Source: Front Public Health. 2021 Jul 28;9:695139. doi: 10.3389/fpubh.2021.695139 (PMC8356061; doi:10.3389/fpubh.2021.695139)
Supplement: Supplementary Material — Supplementary References, Figures, Figure 4 High Res, and Tables. [file Data_Sheet_1.zip › Supplementary Figures.pdf]

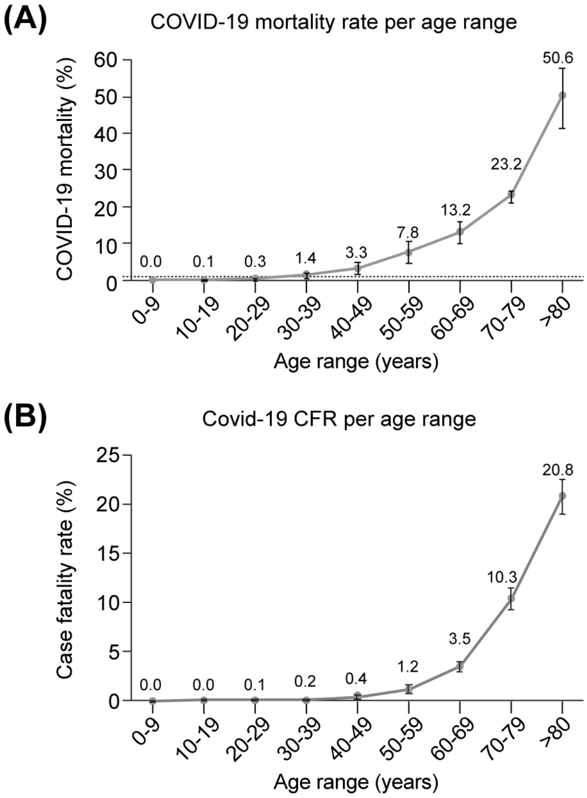

Supplementary Figure 1: (A) Average COVID-19 mortality rate per age range (i.e the percentage of COVID-19 deaths per age range among the total number of COVID-19 deaths). (B) Average COVID-19 case fatality rate (CFR) per age range (i.e the percentage of COVID-19 deaths among the COVID-19 positive cases per age range). Data source are reported in Supplementary Tables 1A and B respectively.

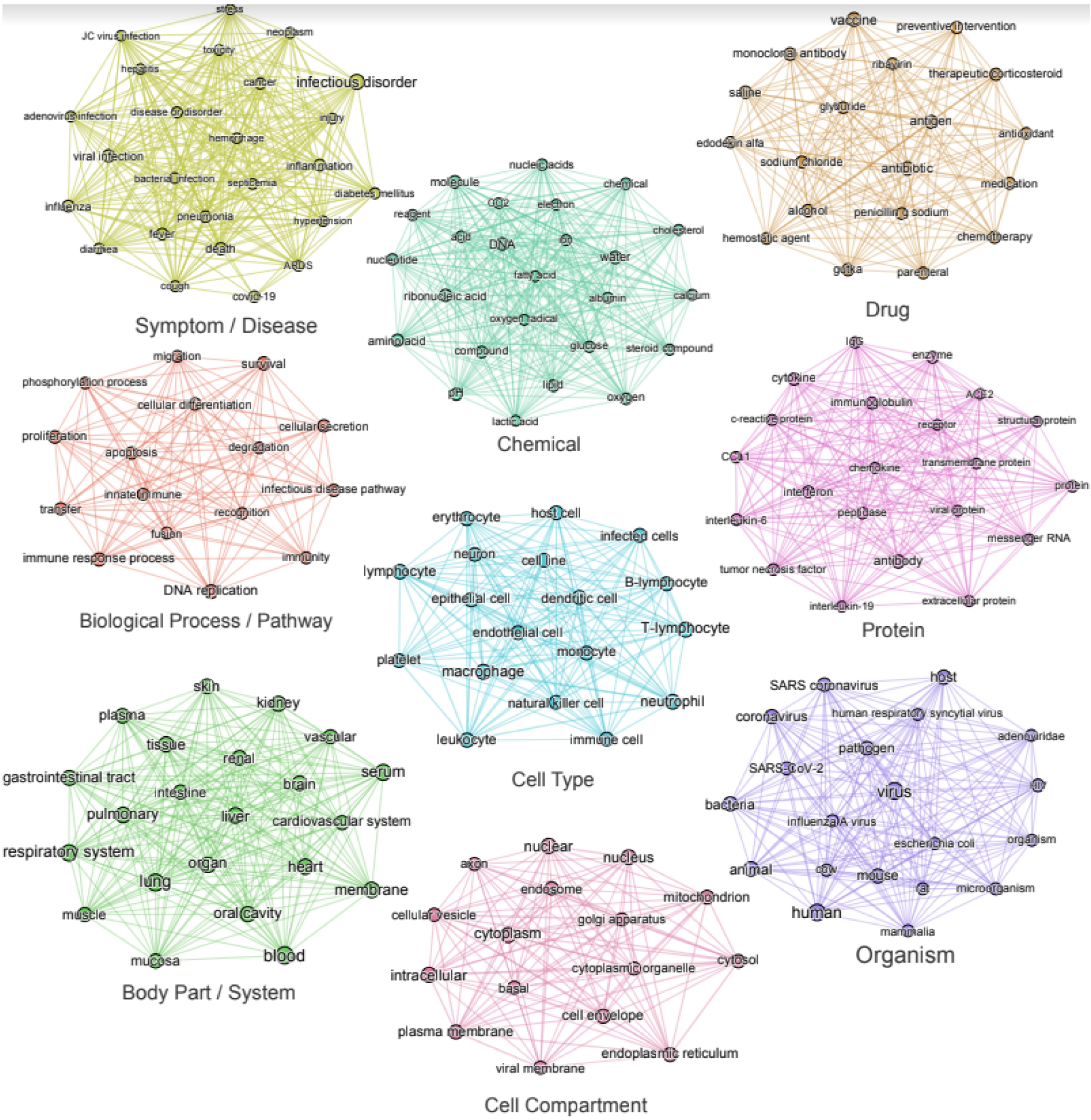

Supplementary Figure 2: Co-mention network overview with zoom on single-entity type subnetworks. Labeled subfigures represent samples of the most frequent entities for each respectively colored single-entity type subnetwork from Figure 2.

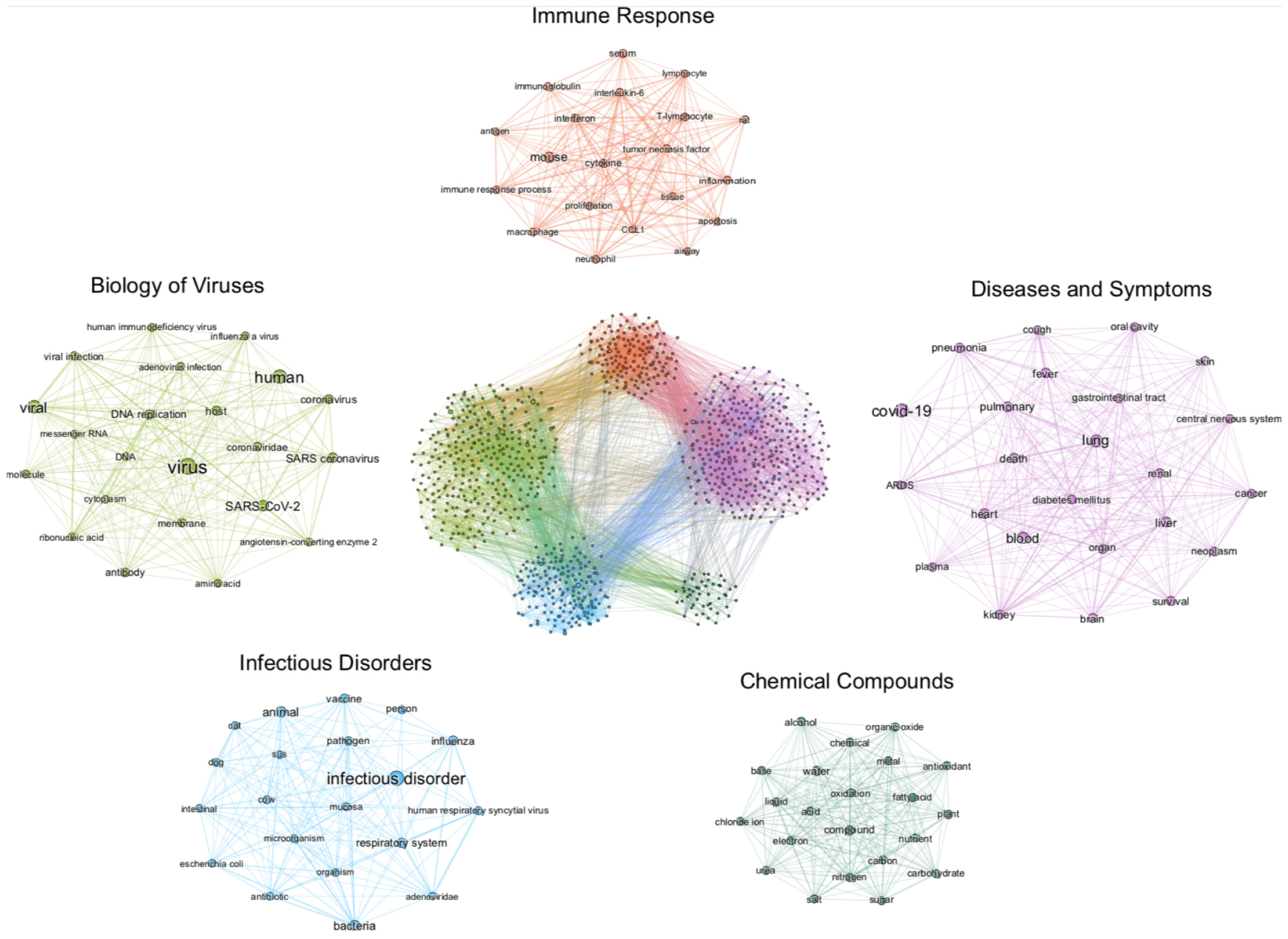

Supplementary Figure 3: Co-mention network communities overview. The figure in the center displays a sample of the knowledge graph containing ~1’000 nodes representing the most frequent entities and edges whose NPMI weight > 0.2. Node sizes are proportional to the weighted degree of nodes, node colors represent different communities detected using the Louvain algorithm and edge thickness is proportional to the NPMI weight of a given entity co-occurrence. Labeled subfigures represent samples of the most frequent entities for each respectively colored community.

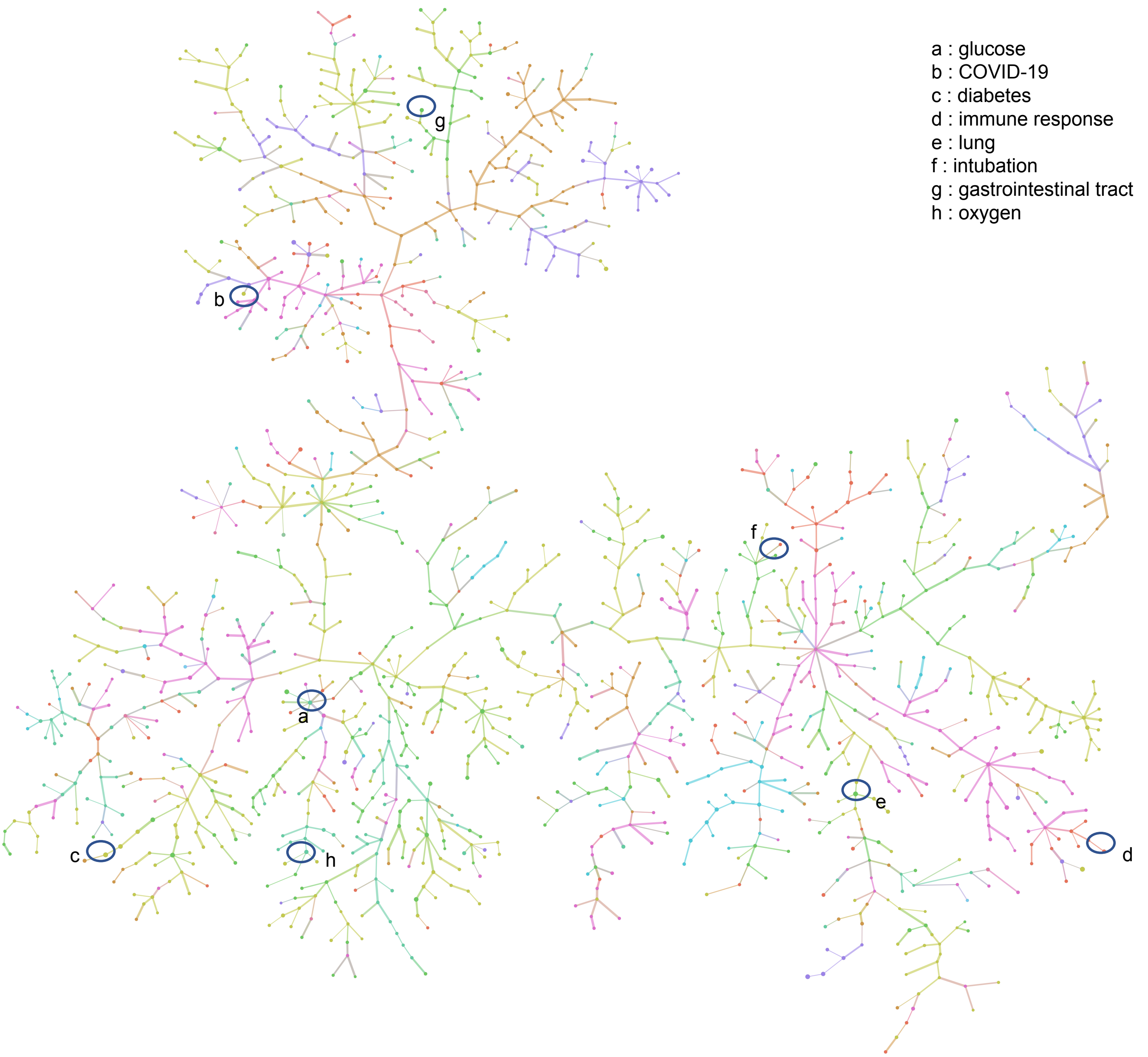

Supplementary Figure 4: Spanning-tree constructed from the 1,500-most frequent entities extracted from the 3,000-most relevant papers in the CORD-19v47 database, according to the query "Glucose is a risk factor for COVID-19". Positions of few key entities are indicated. A high resolution pdf version is available (Figure S4 high res).

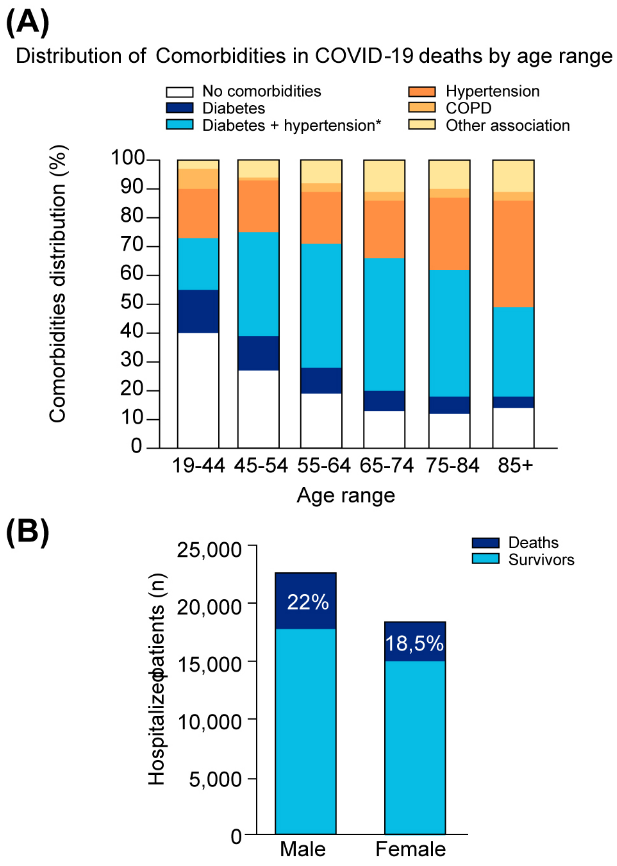

Supplementary Figure 5: (A) Prevalence of comorbidities among COVID-19 related deaths. (B) Distribution of death among total case of hospitalization according to gender. Data are from US populations as of April 30, 2020 and detailed in Supplementary Tables 3A and B respectively.

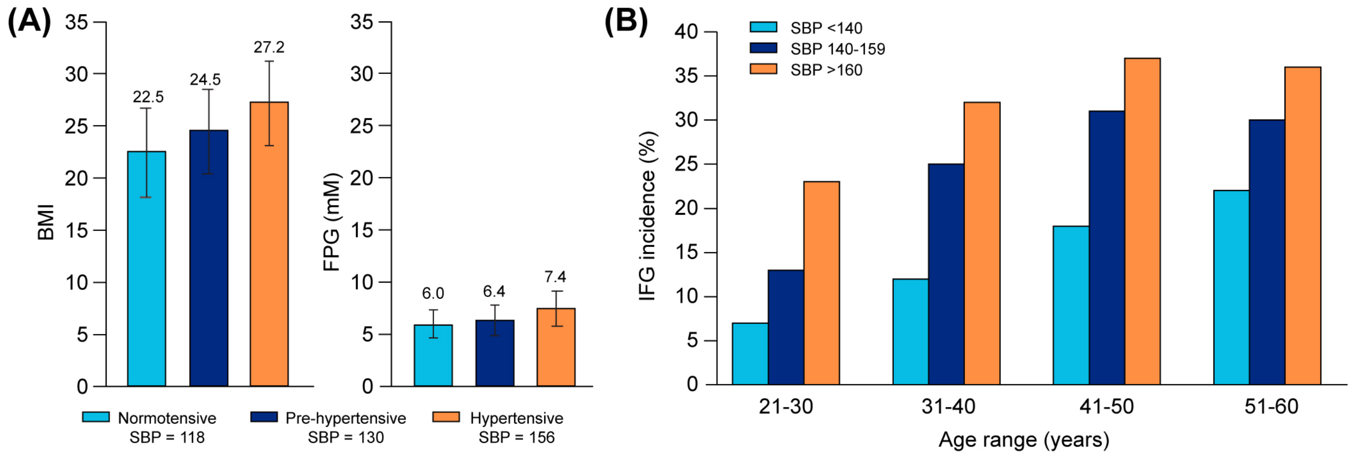

Supplementary Figure 6: Blood glucose in hypertension. (A) Correlation between hypertensive status according to SBP value (SBP=systolic blood pressure in mmHg), and BMI (top) or FPG (bottom). Adapted from Agrawal et al, 2017. (B) Prevalence of impaired fasting glucose (IFG) according to age range and SBP (mmHg). Adapted from Henry et al, 2002 (see Supplementary References)

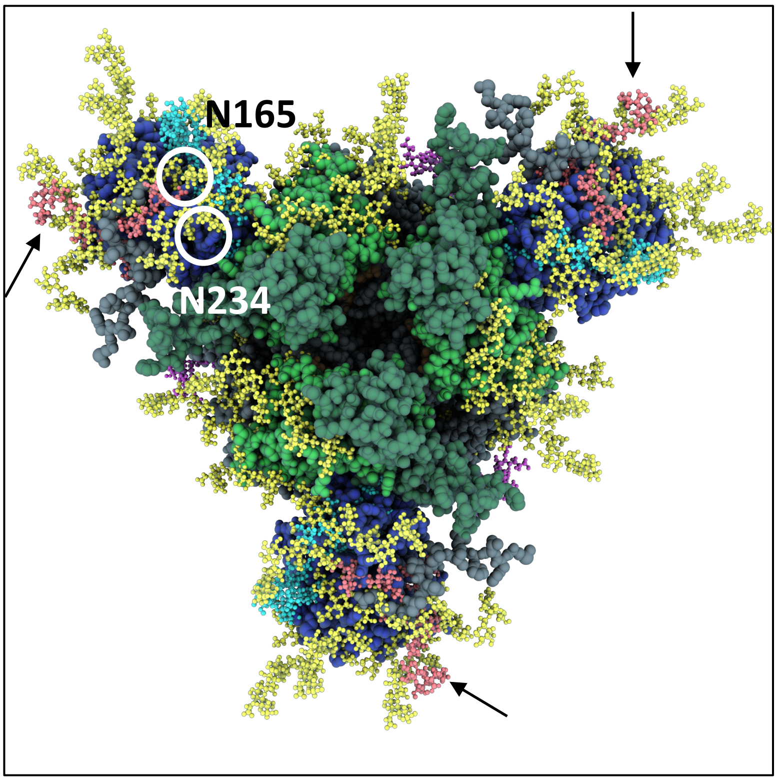

Supplementary Figure 7: Position of the potential new glycan site on the SARS-CoV-2 P.1 variant. The additionnal glycan-tree at N20 is colored in red and indicated with arrows on each S monomer of the spike. Position of N165 and N234 glycan-sites are circled on one single monomer.

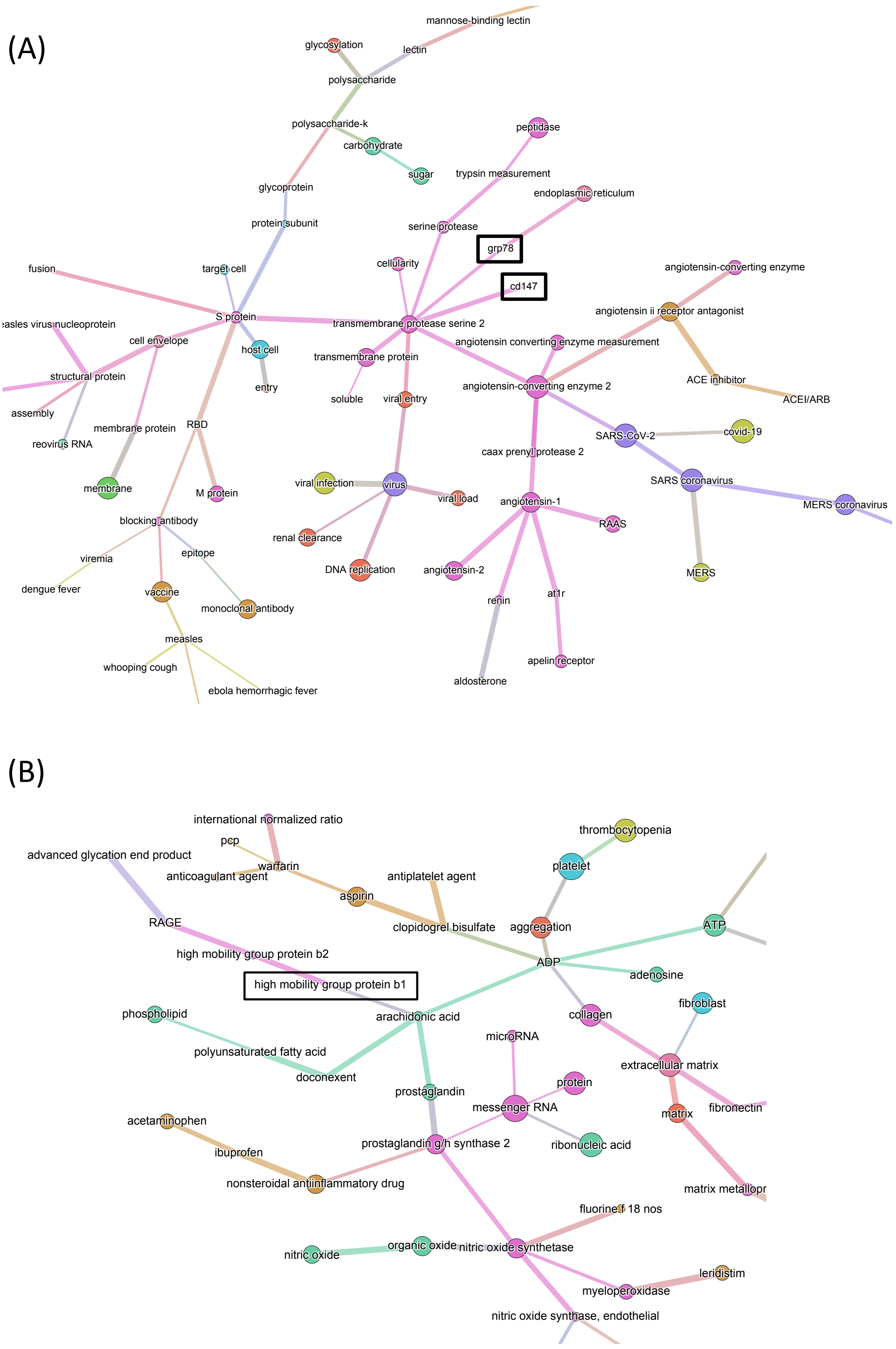

Supplementary Figure 8: The missing entities GRP78, CD147 and HMGB1 were manually imposed on the spanning-tree constructed after the search for “Glucose is a risk factor for COVID-19”. GRP78, CD147 appear in the context of SARS-CoV-2 receptors (A), and HMGB1 appears in the branch containing its receptor RAGE (B), validating the AI-based graph’s construction.
