# Supplementary material for: A Machine-Generated View of the Role of Blood Glucose Levels in the Severity of COVID-19
Source: Front Public Health. 2021 Jul 28;9:695139. doi: 10.3389/fpubh.2021.695139 (PMC8356061; doi:10.3389/fpubh.2021.695139)
Supplement: Supplementary Material — Supplementary References, Figures, Figure 4 High Res, and Tables. [file Data_Sheet_1.zip › Supplementary References.docx]

**Supplementary References**

**References Supplementary Table 1A:** (1–10)

**References Supplementary Table 1B:** (5, 11, 12)

**References Supplementary Tables 4:** (13–18)

**References Supplementary Tables 5:** (19–25)

1. La répartition par âge des décès liés au coronavirus ressemble à une mortalité classique - Libération, https://www.liberation.fr/direct/element/la-repartition-par-age-des-deces-lies-au-coronavirus-ressemble-a-une-mortalite-classique_112144/

2. • Sweden: coronavirus deaths by age | Statista, https://www.statista.com/statistics/1107913/number-of-coronavirus-deaths-in-sweden-by-age-groups/

3. • Spain: coronavirus mortality rate by age 2020 | Statista, https://www.statista.com/statistics/1105596/covid-19-mortality-rate-by-age-group-in-spain-march/

4. • South Korea: coronavirus death cases by age 2020 | Statista, https://www.statista.com/statistics/1105080/south-korea-coronavirus-deaths-by-age/

5. • Poland: Coronavirus (COVID-19) fatalities by age 2020 | Statista, https://www.statista.com/statistics/1110890/poland-coronavirus-covid-19-fatalities-by-age/

6. • Netherlands: coronavirus deaths by age 2021 | Statista, https://www.statista.com/statistics/1109459/coronavirus-death-casulaties-by-age-in-netherlands/

7. • Italy: coronavirus death rate by age | Statista, https://www.statista.com/statistics/1106372/coronavirus-death-rate-by-age-group-italy/

8. • COVID-19 deaths by age and gender Ukraine 2020 | Statista, https://www.statista.com/statistics/1109638/covid-19-deaths-by-age-and-gender-ukraine/

9. • Coronavirus (COVID-19) deaths in Switzerland by age group 2021 | Statista, https://www.statista.com/statistics/1110092/coronavirus-covid-19-deaths-age-group-switzerland/

10. • Coronavirus (COVID-19) deaths by gender and age Germany 2021 | Statista, https://www.statista.com/statistics/1105512/coronavirus-covid-19-deaths-by-gender-germany/

11. What we know about the victims of the coronavirus pandemic in Switzerland - The Local, https://www.thelocal.ch/20200409/what-we-know-about-the-victims-of-the-coronavirus-pandemic-in-switzerland

12. M Roser; H Ritchie; E Ortiz-Ospina; J Hasell. Coronavirus Pandemic (COVID-19), https://ourworldindata.org/coronavirus

13. Y Yang; L Gao; D-J Wang; F-R Li; Y-H Yan; X-Y Wang; X Liao; Q Huang; H Zhang; Q-R Chen; Q Wang; Y Weng; M-H Song. Comparison of 25-hydroxy vitamin D3, adiponectin and apolipoprotein A5 levels in subjects with different glucose tolerance and their correlation with blood lipid. *Biomed Res* 25, 5 (2014)

14. R Tarray; S Saleem; D Afroze; I Yousuf; A Gulnar; B Laway; S Verma. Role of insulin resistance in essential hypertension. *Cardiovascular Endocrinology & Metabolism* 3, 129–133 (2014)

15. Y Mittal. Fasting Blood Glucose Level in Patients Suffering From Hypertension. *Asian Journal of Biomedical and Pharmaceutical Sciences* 4, 4 (2014)

16. Y Heianza; Y Arase; S Kodama; SD Hsieh; H Tsuji; K Saito; S Hara; H Sone. Fasting glucose and HbA1c levels as risk factors for the development of hypertension in Japanese individuals: Toranomon hospital health management center study 16 (TOPICS 16). *J Hum Hypertens* 29, 254–259 (2015)

17. P Duraisamy; S Elango; VP Vishwanandha; R Balamurugan. Prevalence of Mitochondrial tRNA Gene Mutations and Their Association with Specific Clinical Phenotypes in Patients with Type 2 Diabetes Mellitus of Coimbatore. *Genetic Testing and Molecular Biomarkers* 14, 49–55 (2010)

18. B Bhowmik; T Siddiquee; A Mujumder; T Ahmed; H Mahtab; AKA Khan; A Hussain; G Holmboe‑Ottesen; TK Omsland. Diabetes Risk Score for Identifying Cardiometabolic Risk Factors in Adult Bangladeshi Population. *Journal of Diabetology* 9, 8 (2018)

19. S-W Yi; S Park; Y-H Lee; H-J Park; B Balkau; J-J Yi. Association between fasting glucose and all-cause mortality according to sex and age: a prospective cohort study. *Sci Rep* 7, 8194 (2017)

20. AP Yates; I Laing. Age-related increase in haemoglobin A1c and fasting plasma glucose is accompanied by a decrease in β cell function without change in insulin sensitivity: evidence from a cross-sectional study of hospital personnel. *Diabetic Medicine* 19, 254–258 (2002)

21. AI Yashin; SV Ukraintseva; KG Arbeev; I Akushevich; LS Arbeeva; AM Kulminski. Maintaining physiological state for exceptional survival: What is the normal level of blood glucose and does it change with age? *Mech Ageing Dev* 130, 611–618 (2009)

22. GT Ko; HP Wai; JS Tang. Effects of Age on Plasma Glucose Levels in Non-diabetic Hong Kong Chinese. *Croat Med J* 47, 709–713 (2006)

23. D Elahi; D Muller. Carbohydrate metabolism in the elderly. *Eur J Clin Nutr* 54, S112–S120 (2000)

24. D Elahi; DC Muller; SP Tzankoff; R Andres; JD Tobin. Effect of Age and Obesity on Fasting Levels of Glucose, Insulin, Glucagon, and Growth Hormone in Man. *Journal of Gerontology* 37, 385–391 (1982)

25. H Shimokata; DC Muller; JL Fleg; J Sorkin; AW Ziemba; R Andres. Age as independent determinant of glucose tolerance. *Diabetes* 40, 44–51 (1991)
