# Supplementary material for: A Machine-Generated View of the Role of Blood Glucose Levels in the Severity of COVID-19
Source: Front Public Health. 2021 Jul 28;9:695139. doi: 10.3389/fpubh.2021.695139 (PMC8356061; doi:10.3389/fpubh.2021.695139)
Supplement: Supplementary Material — Supplementary References, Figures, Figure 4 High Res, and Tables. [file Data_Sheet_1.zip › Supplementary Tables.docx]

A

| Age range | South Korea | Germany | South Africa | Sweden | Italy | Switzerland | Japan | Ukraine | **Global** | *SEM* |
| --- | --- | --- | --- | --- | --- | --- | --- | --- | --- | --- |
| **0-9** | 0.00 | 0.04 | 0.20 | 0.04 | 0.01 | 0.02 | 0.00 | 0.00 | **0.04** | *+/-0.02* |
| **10-19** | 0.00 | 0.02 | 0.30 | 0.01 | 0.01 | 0.00 | 0.00 | 0.80 | **0.14** | *+/-0.10* |
| **20-29** | 0.00 | 0.12 | 1.30 | 0.16 | 0.05 | 0.00 | 0.10 | 0.30 | **0.25** | *+/-0.15* |
| **30-39** | 0.40 | 0.24 | 6.10 | 0.30 | 0.22 | 0.13 | 0.31 | 3.60 | **1.41** | *+/-0.78* |
| **40-49** | 0.80 | 0.79 | 12.60 | 0.73 | 0.87 | 0.29 | 1.12 | 9.40 | **3.33** | *+/-1.70* |
| **50-59** | 4.59 | 3.08 | 24.40 | 2.41 | 3.40 | 1.58 | 3.26 | 19.90 | **7.83** | *+/-3.17* |
| **60-69** | 11.98 | 8.47 | 26.20 | 6.44 | 9.76 | 5.98 | 9.31 | 27.70 | **13.23** | *+/-3.07* |
| **70-79** | 31.74 | 20.91 | 17.70 | 20.94 | 25.25 | 20.32 | 26.29 | 22.40 | **23.19** | *+/-1.56* |
| **80+** | 50.50 | 66.29 | 11.30 | 68.93 | 60.43 | 71.69 | 59.61 | 15.80 | **50.57** | *+/-8.41* |
| **total** | **100.01** | **99.96** | **100.10** | **99.96** | **100.00** | **100.01** | **100.00** | **99.90** | **99.99** |  |

B

| Age range | South Korea | China | Spain | Italy | Switzerland | Poland | **Global** | *SEM* |
| --- | --- | --- | --- | --- | --- | --- | --- | --- |
| **0-9** | 0.00 | 0.00 | 0.01 | 0.00 | 0.00 | 0.00 | **0.00** | *+/-0.002* |
| **10-19** | 0.00 | 0.20 | 0.02 | 0.00 | 0.00 | 0.05 | **0.05** | *+/-0.032* |
| **20-29** | 0.00 | 0.20 | 0.12 | 0.00 | 0.00 | 0.00 | **0.05** | *+/-0.035* |
| **30-39** | 0.05 | 0.20 | 0.32 | 0.10 | 0.12 | 0.15 | **0.16** | *+/-0.039* |
| **40-49** | 0.10 | 0.40 | 1.06 | 0.20 | 0.08 | 0.30 | **0.36** | *+/-0.149* |
| **50-59** | 0.42 | 1.30 | 3.22 | 0.60 | 0.65 | 1.00 | **1.20** | *+/-0.424* |
| **60-69** | 1.27 | 3.60 | 5.00 | 3.00 | 3.35 | 4.50 | **3.45** | *+/-0.532* |
| **70-79** | 6.68 | 8.00 | 14.30 | 10.20 | 11.59 | 11.20 | **10.33** | *+/-1.109* |
| **80+** | 19.45 | 14.80 | 21.10 | 20.90 | 28.45 | 20.05 | **20.79** | *+/-1.798* |

**Supplementary Tables 1 (Related to Supplementary Figures 1): A)** COVID-19 mortality rate (MR) per age range as of June 2020 in eight different countries. Global rates are plotted in Supplementary Figure 1A. **B)** COVID-19 case fatality rate (CFR) per age range as of June 2020 in six different countries. Global rates are plotted in Supplementary Figure 1B. References are listed in Supplementary References.

A

| **Rank** | **Entity** | **Frequency** | **Entity type** | **Rank** | **Entity** | **Frequency** | **Entity type** |
| --- | --- | --- | --- | --- | --- | --- | --- |
| 1 | COVID-19 | 110,145 | Symptom / Disease | 51 | amino acid | 14,227 | Chemical |
| 2 | virus | 75,012 | Organism | 52 | pH | 14,107 | Chemical |
| 3 | infectious disorder | 73,574 | Symptom / Disease | 53 | gastrointestinal tract | 13,510 | Organ / System |
| 4 | coronavirus | 67,945 | Organism | 54 | immunoglobulin | 13,198 | Protein |
| 5 | human | 61,816 | Organism | 55 | antibiotic | 13,034 | Drug |
| 6 | viral | 52,801 | Organism | 56 | outbreak | 12,766 | Symptom / Disease |
| 7 | SARS-COV-2 | 49,386 | Organism | 57 | cow | 12,656 | Organism |
| 8 | SARS coronavirus | 41,463 | Organism | 58 | transfer | 12,625 | Biological Process / Pathway |
| 9 | blood | 33,970 | Organ / System | 59 | tissue | 12,542 | Organ / System |
| 10 | person | 33,893 | Organism | 60 | nucleic acids | 12,530 | Chemical |
| 11 | coronaviridae | 32,976 | Organism | 61 | ARDS | 12,392 | Symptom / Disease |
| 12 | animal | 31,998 | Organism | 62 | brain | 12,307 | Organ / System |
| 13 | lung | 31,752 | Organ / System | 63 | cardiovascular system | 11,864 | Organ / System |
| 14 | respiratory system | 31,221 | Organ / System | 64 | interferon | 11,687 | Protein |
| 15 | death | 31,114 | Symptom / Disease | 65 | skin | 11,686 | Organ / System |
| 16 | host | 27,219 | Organism | 66 | renal | 11,630 | Organ / System |
| 17 | bacteria | 26,082 | Organism | 67 | human respiratory syncytial virus | 11,356 | Organism |
| 18 | influenza | 26,000 | Symptom / Disease | 68 | T-lymphocyte | 11,189 | Cell Type |
| 19 | antibody | 24,447 | Protein | 69 | plasma | 11,132 | Organ / System |
| 20 | fever | 24,295 | Symptom / Disease | 70 | MERS coronavirus | 11,063 | Organism |
| 21 | vaccine | 24,073 | Drug | 71 | disease or disorder | 11,035 | Symptom / Disease |
| 22 | pneumonia | 23,998 | Symptom / Disease | 72 | compound | 10,907 | Chemical |
| 23 | viral infection | 23,797 | Symptom / Disease | 73 | nucleotide | 10,780 | Chemical |
| 24 | pathogen | 23,441 | Organism | 74 | neoplasm | 10,750 | Symptom / Disease |
| 25 | mouse | 23,122 | Organism | 75 | enzyme | 10,567 | Protein |
| 26 | heart | 22,583 | Organ / System | 76 | cytoplasm | 10,364 | Cell Compartment |
| 27 | DNA replication | 21,577 | Biological Process / Pathway | 77 | lower | 10,342 | Organ / System |
| 28 | pulmonary | 21,261 | Organ / System | 78 | nuclear | 10,305 | Cell Compartment |
| 29 | serum | 20,300 | Organ / System | 79 | antigen | 10,263 | Drug |
| 30 | water | 19,746 | Chemical | 80 | immunity | 10,236 | Biological Process / Pathway |
| 31 | survival | 18,915 | Biological Process / Pathway | 81 | peptidase | 10,156 | Protein |
| 32 | DNA | 16,948 | Chemical | 82 | adenoviridae | 10,148 | Organism |
| 33 | infectious disease pathway | 16,882 | Biological Process / Pathway | 83 | adenovirus infection | 10,111 | Symptom / Disease |
| 34 | membrane | 16,708 | Organ / System | 84 | hypertension | 1,094 | Symptom / Disease |
| 35 | liver | 16,688 | Organ / System | 85 | lymphocyte | 10,064 | Cell Type |
| 36 | inflammation | 16,554 | Symptom / Disease | 86 | diarrhea | 10,039 | Symptom / Disease |
| 37 | oral cavity | 16,522 | Organ / System | 87 | macrophage | 10,028 | Cell Type |
| 38 | cough | 16518 | Symptom / Disease | 88 | proliferation | 10,006 | Biological Process / Pathway |
| 39 | ribonucleic acid | 16,397 | Chemical | 89 | ACE2 | 9,933 | Protein |
| 40 | cancer | 15,767 | Symptom / Disease | 90 | neutrophil | 9,920 | Cell Type |
| 41 | human immunodeficiency virus | 15,733 | Organism | 91 | interleukin-6 | 9,883 | Protein |
| 42 | organ | 15,679 | Organ / System | 92 | nasopharyngeal | 9,851 | Symptom / Disease |
| 43 | kidney | 15,600 | Organ / System | 93 | microorganism | 9,808 | Organism |
| 44 | cytokine | 15,584 | Protein | 94 | airway | 9,703 | Organ / System |
| 45 | immune response process | 15,014 | Biological Process / Pathway | 95 | child | 9,650 | Organism |
| 46 | diabetes mellitus | 14,984 | Symptom / Disease | 96 | mammalia | 9,643 | Organism |
| 47 | molecule | 14,846 | Chemical | 97 | messenger RNA | 9,614 | Protein |
| 48 | influenza A virus | 14,686 | Organism | 98 | eye | 9,588 | Organ / System |
| 49 | SARS | 14,372 | Symptom / Disease | 99 | respiratory system disorder | 9,528 | Symptom / Disease |
| 50 | oxygen | 14,289 | Chemical | 100 | injury | 9,438 | Symptom / Disease |

B

| **Rank** | **Entity** | **Rank** | **Entity type** |
| --- | --- | --- | --- |
| 1 | water | 10 | nucleotide |
| 2 | DNA | 11 | viral RNA |
| 3 | RNA | 12 | chemical |
| 4 | molecule | 13 | lipid |
| 5 | oxygen | 14 | reagent |
| 6 | amino acid | 14 | acid |
| 7 | pH | 16 | CO2 |
| 8 | nucleic acids | 17 | glucose |
| 9 | compound | … |  |

**Supplementary Tables 2 (Related to Table 1): A)** Ranking of the 100 most frequently mentioned terms in the CORD-19v47 database following COVID-19-related entity types recognition. (i.e. ‘COVID-19’ is mentioned in 110,145 distinct papers). **B)** Ranking of top mentioned terms from the entity type “*chemical*” in the CORD-19v47 database.

Note that in order to filter out mentions of glucose as a chemical appearing in experimental procedures, we have excluded sections entitled 'Methods' and 'Materials and Methods' from our analysis.

A

| **Age range** | **0-18** | **19-44** | **45-54** | **55-64** | **65-74** | **75-84** | **85+** |
| --- | --- | --- | --- | --- | --- | --- | --- |
| Total death n | 0 | 241 | 468 | 1,181 | 2,010 | 2,356 | 2,087 |
| no comorbidities n (%) | 0 | 97 (40) | 126 (27) | 225 (19) | 261 (13) | 283 (12) | 292 (14) |
| Diabetes n (%) | 0 | 36 (15) | 56 (12) | 106 (9) | 141 (7) | 141 (6) | 84 (4) |
| Diabetes + Hypertension* n (%) | 0 | 43 (18) | 169 (36) | 508 (43) | 925 (46) | 1036 (44) | 647 (31) |
| Hypertension n (%) | 0 | 41 (17) | 84 (18) | 213 (18) | 402 (20) | 589 (25) | 772 (37) |
| COPD n (%) | 0 | 17 (7) | 5 (1) | 35 (3) | 60 (3) | 71 (3) | 63 (3) |
| Other combinations n (%) | 0 | 7 (3) | 28 (6) | 94 (8) | 221 (11) | 236 (10) | 229 (11) |

B

|  | Male | Female |
| --- | --- | --- |
| Total case (n) | 22,566 | 18,441 |
| Total death (n) (%) | 4,937 (22%) | 3,406 (18,5%) |

**Supplementary Tables 3 (Related to Supplementary Figures 5). (A)** Prevalence of comorbidities among COVID-19 related deaths as plotted in Supplementary Figure 5A. (**B)** Distribution of death among total cases of hospitalization according to gender as plotted in Supplementary Figure 5B. Data from US populations as of April 30, 2020. Data source: <https://ehrn.org/prevalence-of-comorbidities-in-covid-19-related-hospitalizations-and-deaths/>

**A**

|  | FPG (mM) | | 2h PPG (mM) | | HbA1c (%) | |
| --- | --- | --- | --- | --- | --- | --- |
|  | Control | T2DM | Control | T2DM | Control | T2DM |
| Duraisamy et al, 2010 | 4.5 | 9.01 | 5.32 | 13.05 | 5.22 | 8.97 |
| Bhowmik et al, 2018 | 4.7 | 9.5 | 5.4 | 13.9 | 5.3 | 7.6 |
| Yang et al, 2014 | 5.26 | 9.79 | 5.45 | 14.53 | 4.23 | 8.19 |
| **Mean** | **4.82** | **9.4** | **5.4** | **13.8** | **4.92** | **8.3** |
| *SD* | *+/-0.4* | *+/-0.4* | *+/-0.06* | *+/-0.75* | *+/-0.6* | *+/-0.7* |

**B**

|  | Control | | Hypertensive | |
| --- | --- | --- | --- | --- |
|  | FPG mM | *n* | FPG mM | *n* |
| Mittal, 2014 | 4.6 | *50* | 5.47 | *70* |
| Tarray et al, 2014 | 4.92 | *50* | 5.31 | *50* |
| Heianza et al, 2015 | 5.24 | *8,486* | 5.44 | *1,098* |
| **Mean** | **4.92** |  | **5.41** |  |
| *SD* | *+/-0.32* |  | *+/-0.08* |  |

**Supplementary Tables 4 (Related to Figures 9): (A)** Values of FPG, 2h-PPG and HbA1c in control (n=2,116) versus diabetic patients (n=439) according to values reported in 3 different studies. Mean values are plotted in Figure 9A. (**B)** Values of FPG in control (n=8,586) versus hypertensive patients (n=1,218) according to values reported in three different studies. Mean values are plotted in Figure 9B. References are listed in Supplementary References.

A

| Age | FPG (mM) | | | | **Mean** | *SEM* |
| --- | --- | --- | --- | --- | --- | --- |
| <19 | 5.2 | NA | 4.9 | 4.78 | **4.96** | *+/-0.125* |
| 20-29 | 5.1 | NA | 5 | 4.83 | **4.98** | *+/-0.08* |
| 30-39 | 5.05 | 4.44 | 5.1 | 4.97 | **4.89** | *+/-0.15* |
| 40-49 | 5.5 | 4.61 | 5.15 | 5.22 | **5.12** | *+/-0.19* |
| 50-59 | 5.7 | 4.78 | 5.2 | 5.44 | **5.28** | *+/-0.19* |
| 60-69 | 5.7 | 5.06 | 5.3 | 5.50 | **5.39** | *+/-0.14* |
| 70-79 | 5.71 | 5.39 | NA | 5.56 | **5.55** | *+/-0.09* |
| 80-89 | 5.72 | 5.72 | NA | 5.64 | **5.69** | *+/-0.03* |
| >90 | 5.72 | 6.06 | NA | NA | **5.89** | *+/-0.17* |
|  | Ko et al, 2006 *(n=6,901)* | Yashin et al, 2009 *(n= 5,128)* | Yates and Laing, 2002 *(n=191)* | Yi et al, 2017 *(n>10,000)* |  |  |

B

| Age | FPG in male (mM) | | **Mean FPG male** | FPG in female (mM) | | **Mean FPG female** |
| --- | --- | --- | --- | --- | --- | --- |
| <19 | NA | 4.78 | **4.78** | NA | 4.78 | **4.78** |
| 20-29 | NA | 4.89 | **4.89** | NA | 4.78 | **4.78** |
| 30-39 | 4.44 | 5.11 | **4.78** *+/-0.33* | 4.39 | 4.89 | **4.64** *+/-0.25* |
| 40-49 | 4.58 | 5.39 | **4.99** *+/-0.40* | 4,53 | 5.03 | **4.78** *+/-0.25* |
| 50-59 | 4.78 | 5.56 | **5.17** *+/-0.39* | 4.72 | 5.22 | **4.97** *+/-0.25* |
| 60-69 | 5.17 | 5.56 | **5.36** *+/-0.19* | 5.03 | 5.42 | **5.22** *+/-0.19* |
| 70-79 | 5.67 | 5.56 | **5.61** *+/-0.05* | 5.42 | 5.56 | **5.49** *+/-0.07* |
| 80-89 | 5.78 | 5.61 | **5.69** *+/-0.08* | 5.78 | 5.67 | **5.72***+/-0.05* |
| >90 | 6.11 | NA | **6.11** | 6.33 | NA | **6.33** |
|  | Yashin et al, 2009 *(n= 2,336)* | Yi et al, 2017 *(n>10,000)* |  | Yashin et al, 2009 *(n=2,873)* | Yi et al, 2017 *(n>10,000)* |  |

C

| Age | 2h PPG (mM) | | | | **Mean** | **SEM** |
| --- | --- | --- | --- | --- | --- | --- |
| 20-29 | 6.11 | 7.22 | 7.28 | NA | **6.87** | *+/-0.38* |
| 30-39 | 6.22 | 7.44 | 7.61 | 5.9 | **6.79** | *+/-0.43* |
| 40-49 | 6.78 | 7.67 | 8.00 | 6.2 | **7.16** | *+/-0.41* |
| 50-59 | 7.22 | 7.22 | 8.39 | 6.5 | **7.33** | *+/-0.39* |
| 60-69 | 7.78 | 8.44 | 8.72 | 7.1 | **8.01** | *+/-0.36* |
| 70-79 | 7.78 | 8.39 | 9.61 | 8 | **8.44** | *+/-0.41* |
| 80-89 | NA | 9.22 | 10.10 | 8.4 | **9.24** | *+/-0.49* |
|  | Chia et al, 2018 *(n=2,777)* | Elahi et al, 1982 *(n=186)* | Elahi et al, 2000 *(n=NA)* | Shikomata et al, 1991 *(n=742)* |  |  |

**Supplementary Tables 5 (Related to Figures 8): (A)** Values of FPG in function of age range according to data reported in 4 different studies. Mean values are plotted in Figure 8A. **(B)** Values of FPG in function of age range and gender according to data reported in 2 different studies. Mean values are plotted in Figure 8B. **(C)** Values of 2h PPG after an OGTT test in function of age range according to values reported in 4 different studies. Mean values are plotted in Figure 8C. The number of participants (n) in each study is reported. In some cases, absolute values are not reported and then directly extracted from raw plots. All references are available in the manuscript or in Supplementary References.
