# Supplementary figures and images for: A Machine-Generated View of the Role of Blood Glucose Levels in the Severity of COVID-19
Source: Front Public Health. 2021 Jul 28;9:695139. doi: 10.3389/fpubh.2021.695139 (PMC8356061; doi:10.3389/fpubh.2021.695139)

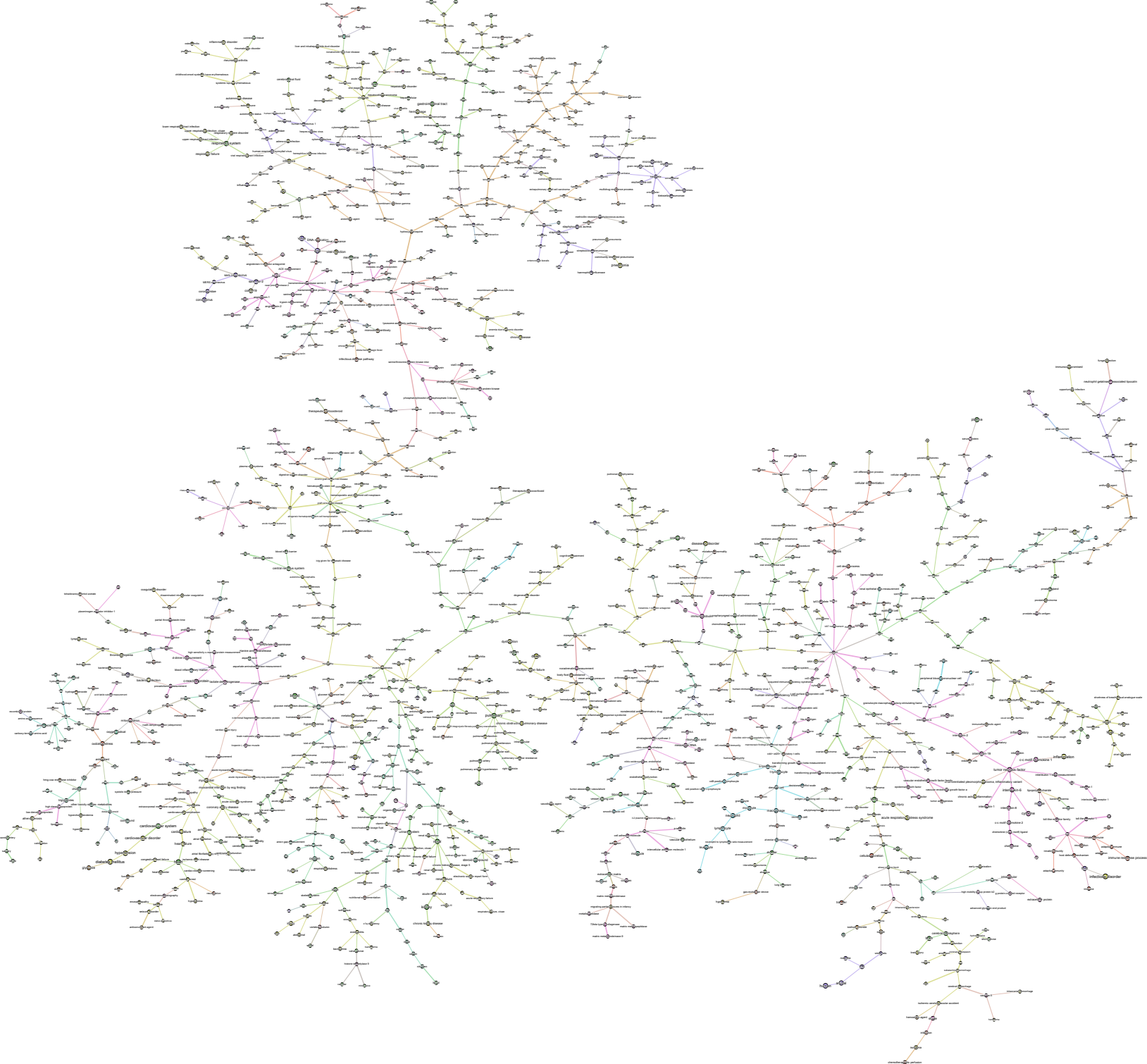

Supplement: Supplementary Material — Supplementary References, Figures, Figure 4 High Res, and Tables. [file Data_Sheet_1.zip › Supplementary Figure 4 high-res.pdf]
